# Perspectives of women from migrant and refugee backgrounds accessing the Cross Cultural Worker Service in maternity and early childhood services—a qualitative study

**DOI:** 10.3389/fgwh.2025.1553677

**Published:** 2025-03-06

**Authors:** Helen J. Rogers, Caroline S. E. Homer, Amanda Henry

**Affiliations:** ^1^ Discipline of Women's Health, School of Clinical Medicine, University of New South Wales, Kensington, NSW, Australia; ^2^ Burnet Institute, Melbourne, VIC, Australia; ^3^ Faculty of Health, University of Technology Sydney, Sydney, NSW, Australia; ^4^ The George Institute for Global Health, UNSW Medicine and Health, Sydney, NSW, Australia; ^5^ Department of Women's and Children's Health, St George Hospital, Kogarah, NSW, Australia

**Keywords:** migrants, refugees, perinatal, bicultural worker, model of care, culturally responsive care, access and quality, service navigation

## Abstract

**Introduction:**

Women from migrant and refugee backgrounds living in high-income countries have an increased risk of adverse perinatal outcomes and lower satisfaction with healthcare. A Cross Cultural Workers (CCWs) Service was implemented in Sydney, Australia, supporting women and families throughout pregnancy to their child being 5 years old.

**Methods:**

This study aimed to describe women's experience of the CCW Service and recommendations for improvement using interviews at 6 or 12 months postpartum. A framework approach was used for analysis.

**Results:**

Four themes were generated from 23 interviews; (1) gaining knowledge, (2) strengthening capacity, (3) providing support; and (4) sharing culture, language, and migration journey. The impact of COVID-19 was a cross-cutting issue.

**Discussion:**

The CCW Service was highly regarded, helpful, informative, and enhanced women's care experience. Recommendations for improvement were increased CCW workforce and provision of group education. This model has the potential to improve perinatal care of women from migrant and refugee backgrounds.

## 1 Introduction

Globally, the scale of international migration is increasing (1). In 2020, an estimated 281 million people, 3.6% of the global population living in a country other than their country of birth, an increase from 221 million people (3.2%) since 2010 (1). Approximately 48% of migrants are women, many of whom are of childbearing age (2). Their experiences are key determinants of health and well-being (3).

Women from migrant and refugee backgrounds settling in high-income countries (HIC) may experience significant barriers accessing maternal and child and family health services (3, 4), including cost of services, language barriers, unfamiliarity with healthcare systems, limited health literacy, xenophobia, discrimination, and cultural insensitivity (5). They also face a complex intersection of challenges when settling in a new country, including, new culture, language and systems, securing housing and employment, anxiety about changes in visa conditions, making friends, financial and family responsibilities, which may be compounded by the impact of loss, dislocation, grief, torture and refugee trauma, and cultural dissonance (3, 6). The closure of international borders due to the COVID-19 pandemic particularly impacted women from migrant and refugee communities in the perinatal period with loss of social support networks (7).

Women from migrant and refugee backgrounds experience increased adverse perinatal outcomes compared to women born in the host country. This includes, increased rates of mental health issues (5, 8, 9), lower levels of attendance (5), and later access to antenatal care (10, 11), increased congenital anomalies and preterm birth (3, 8), and higher rates of smaller for gestational age infants (12, 13), stillbirth (13, 14), and infant admission to neonatal care (15, 16). These adverse perinatal outcomes may have a profound impact on child development (17, 18).

Tailored models of care that improve access in order to improve perinatal health outcomes are needed. A systematic scoping review of pregnancy and postpartum models of care for women from migrant and refugee background living in HIC identified potentially effective models including female paraprofessional bicultural/bilingual workers serving as links between health, community-based services, and local communities, to improve access and quality of service provision (19). One such model, the Cross Cultural Workers (CCWs) in Maternity and Child and Family Health Services (the CCW Service), was implemented during 2017 in metropolitan Sydney, Australia. The service supports women and families from migrant, refugee, and asylum seeker backgrounds to access and navigate maternity, child and family health (CFH) services, and community-based organisations through pregnancy, and into early childhood until school entry (the First 2000 Days). The aims of this paper were to (1) describe women's experiences of engaging with the CCW Service, and (2) ascertain recommendations for CCW Service improvement.

## 2 Materials and methods

### 2.1 Design

A qualitative study was undertaken using semi-structured interviews.

### 2.2 Setting

The CCW Service is based in South-Eastern Sydney, Australia, a culturally diverse metropolitan area. There are three publicly-funded maternity facilities with approximately 10,000 births per year total, with 38% of women being born in a non-English speaking country (20). Public antenatal care is offered through midwifery-led and doctor-led clinics, midwifery group practice, group models of antenatal care, or shared care arrangements between a general practitioner (family doctor) and hospital antenatal clinics. Following birth and discharge, women and families have access to universal child and family health nursing services provided from birth until school entry.

The CCW Service is an enhanced model of care provided alongside existing maternity and CFH services. The Service employs three female, part-time, Cross-Cultural Workers (CCWs) with personal lived experience of the migration journey, fluent in both their country-of-origin language and English. This includes CCWs from Nepal (Nepali and Hindi speaking), Bangladesh (Bangla, Hindi and Urdu speaking) and Indonesia (Bahasa speaking). The CCWs support women and families from migrant and refugee backgrounds who are socially isolated, financially disadvantaged, have limited support and/or psychosocial risk factors. Priority is given to women resident in Australia for less than five years and/or Medicare ineligible, although absence of these factors does not exclude women from accessing the Service. This prioritization acknowledges several potential barriers to healthcare access, including the complexities of resettlement, understanding and navigating available services, and managing maternity care costs. These priority areas directly informed our research aims to explore women's experiences of the CCW Service, particularly its acceptability and appropriateness, and to gather recommendations for service improvement.

**Figure 1:**
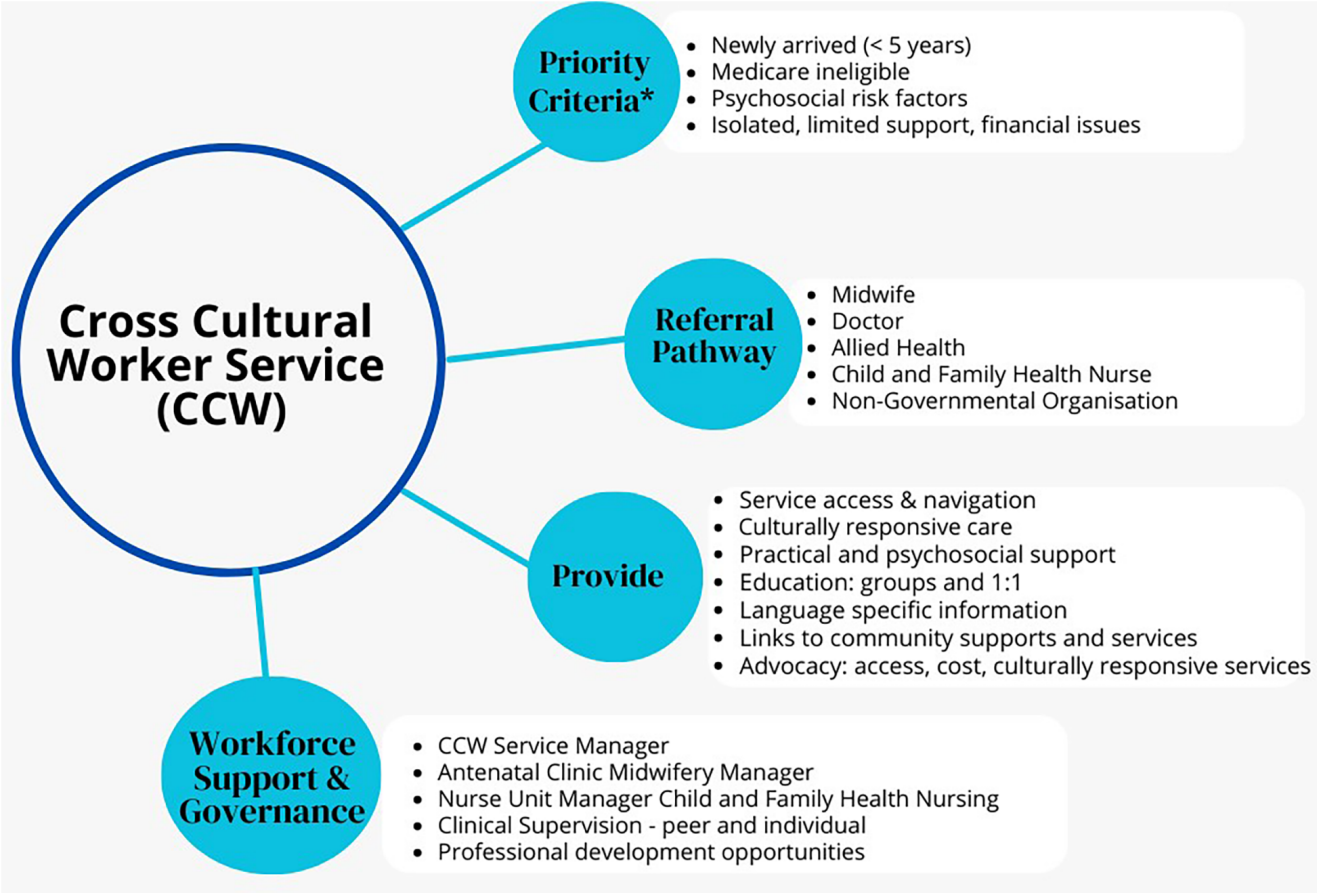
Cross Cultural Worker Service model of care. *All women and families from migrant and refugee backgrounds can access the CCW Service, however priority is given to listed criteria. **Medicare is Australia's publicly-funded universal health insurance scheme. It guarantees all Australians (and some overseas visitors) access to a wide range of health and hospital services at low or no cost (22).

In the Australian context, Medicare is Australia's publicly-funded universal health insurance scheme. Access to publicly-funded maternal health services is free to women who are refugees, seeking asylum, Australian citizens, permanent residents, and New Zealand citizens. The Australian Government recommends all temporary visa holders take out private health insurance, and private health insurance is a prerequisite for some temporary visa types. However, the level of coverage may vary among insurance providers, and a 12-month waiting period for pregnancy-related care may be applied (21). The model of care and inclusion criteria for the CCW Service are detailed in Figure 1.

The role of the CCWs is to provide safe, respectful, client-centred and compassionate care for women and their families across the continuum from pregnancy, labour and birth, postpartum, and the first five years of a child's life. CCWs are part of the multidisciplinary team with midwives, child and family health nurses (CFHN), doctors, allied health and other service providers. The CCW Service provides and evaluates care in partnership with women, their partner and families, referring to and collaborating with other health and social care professionals as needed. Support is tailored according to need and consists of: (a) supporting women and families to navigate maternity, CFH, and community-based services, (b) enabling early access and ongoing service engagement across the continuum of pregnancy and the transition to CFH services, (c) providing culturally appropriate support to women and their families.

CCWs act as cultural brokers, peer support workers, and mediators between women and service providers, to provide practical and psychosocial support, health promotion, health protection, language specific information (when available), education for women and families and service providers, and client advocacy and collaboration with health services, local communities and agencies to build capacity to provide culturally responsive services. The CCWs are co-located in hospitals and community-based clinics. Referrals are received from midwives, doctors, CFHN, allied health and non-governmental organisations (NGO). All client contact occurs during office hours. The number, frequency and type of interaction (including face-to-face, telehealth, telephone, text messaging, and email contact) varies depending on the preference and needs of each woman and family.

### 2.3 Eligibility and recruitment

Recruitment occurred February 2019-July 2021. The CCWs discussed the study during their usual interactions. The CCWs' involvement in recruitment was carefully designed to enhance cultural and emotional safety while preventing any potential coercion. CCWs acted as cultural bridges, helping explain the research process and participants' rights in a culturally responsive way, particularly important for women who may be unfamiliar with research participation. While CCWs facilitated initial contact, several safeguards were implemented to ensure voluntary participation. All participants were provided with a written consent form emphasizing the voluntary, anonymous, and confidential nature of the study. Women were explicitly informed that the CCWs would have no knowledge of their responses, and that their participation decision would not affect their current or future care, nor impact the CCWs' employment. This approach supported meaningful engagement while maintaining clear boundaries between recruitment and participation decisions. The consent form was available in Bangla, Indonesian Bahasa, and Nepali, and interpreter services used as required to explain the research.

In total 224 women consented to participate in the study, involving completion of surveys (results previously published (23), with option to indicate interest in a follow-up interview at 6 or 12 months postpartum. A total of 154 interested women provided their contact details. Purposive sampling was used to ensure representation of the countries of birth, ethnicities, years residence in Australia, maternal age range, and parity. An email invitation was sent by the lead researcher (HJR), in a relevant language as required, providing previously signed study information sheet and consent form to allow for reading in their own time, discussion with friend or family member, without feeling pressured. A total of 37 women were invited to participate in an interview, 23 accepted the invitation.

COVID-19 public health restrictions impacted on the CCW Service provision of care, and recruitment to the study. Interviews commenced in February 2020, however due to redeployment of lead researcher to pandemic response, further interviews were postponed to April 2021. Due to timing of the interviews, women's experience of the COVID 19 pandemic emerged during the interviews.

### 2.4 Data collection

Data collection involved semi-structured telephone interviews. The interview guide was developed by the research team based on a literature search of perinatal health service evaluation by service users, local maternity and CFH services consumer evaluations, as well as clinical and research experience. The questions were reviewed by the CCW Service Working Group and pilot tested with two colleagues and a qualitative researcher (See Table 1).

**Table 1:** Semi-structured interview guide.

Can you tell me about your experience/interaction with the CCW Service?
• during pregnancy • following your baby's birth What did you think of the CCW Service? What were some of the things/services the CCW provided? Example prompt questions:
• Did the CCW help you access any other services? • Did you know about these services and how to access prior to meeting the CCW? Were there any particular aspects of the CCW Service:
• you liked? • did not like? • you wanted more or less of? • you expected but didn’t receive? What could we do differently/better in the future? How do you think your experience would have been if you had not met the CCW?
• Why do you say this? What would you do now if you were concerned about you or your baby's health? Example prompts:
• Where would you go? • What services? • Did you know this prior to seeing the CCW? Would you like to provide any other feedback or ask any questions?

The lead researcher (HJR) telephoned women to introduce herself, provide an overview of the purpose of the study, the interview process, preferred time, and preference for an interpreter. Women had the opportunity to ask questions about the study. Demographic data of all participants was collected, including age, country of birth, ethnicity, period of residence in Australia, main language spoken at home, and parity.

All interviews were facilitated by the lead researcher (HJR) in English. Three interviews required an interpreter (one Spanish and two Thai speaking). Professional interpreters were engaged through an accredited interpreting service and bound by their professional code of ethics and confidentiality agreements. Interpreters were briefed about the research aims and provided with guidelines emphasizing the importance of accurate translation and maintaining participant privacy. Interpreters provided services via telephone rather than in-person, adding another layer of anonymity. All interpreted sessions were conducted in private spaces, and participants were informed of their right to request a different interpreter if they had any concerns about confidentiality.

All interviews were audio recorded with participants' verbal consent and transcribed verbatim. Researcher reflections were immediately documented following each interview as field notes and used in the analysis. No new themes were raised in the last five interviews indicating data saturation, however all data were analysed and included in the study. The interviews lasted 39 min on average (range 20–63 min).

### 2.5 Data analysis

Interviews were transcribed verbatim, de-identified and analysed by HJR before being cross checked by AH and CSEH. Framework analysis (24, 25) was used to analyse interview data. Framework analysis uses a matrix spreadsheet enabling researchers to systematically organise participants and thematic codes, to analyse, compare, and contrast data across and within participants to identify themes (25).

To ensure familiarisation with the data*,* HJR listened to the audio files, reviewed field notes, and read through transcripts repeatedly to gain an overall impression of the data. An inductive, iterative approach of reading the transcripts line by line to select words, sentences and phrases that contained information relevant to the research aims. Microsoft Office Excel (V2306 Build 16529.20182) was used to develop a matrix spreadsheet to allocate codes, categories, themes, and sub-themes. Relationships between categories were inductively derived from the data, based on the research aims (24). These were then merged into main themes after discussion between all authors. Themes and related subthemes have been presented with quotes in *italics* identified by respondent and number, (e.g., W1, where W = women, and 1 = respondent number). Some quotes are the translated words of the interpreter; however, it is clearly indicated when this occurs. Where necessary for fluency, brevity or confidentiality, words have been deleted indicated by an ellipsis (…). In some instances, to improve clarity or protect confidentiality, words have been inserted which are indicated by [square brackets]. The first author of this paper [HJR] currently manages the CCW Service. Reflexive practices to increase awareness of beliefs or assumptions were embedded at each stage of the research process. For example, reflexive activities included journalling, regular reflective supervision, critical enquiry, and contemporaneous feedback processes to progress thinking, analysis, and writing to reduce potential bias.

### 2.6 Ethical approval

Prospective approval by the South Eastern Sydney Local Health District Human Research Ethics Committee in October 2017, approval number: HREC 17/257.

## 3 Results

Of the 23 participating women, 15 were 6 months postpartum and eight 12 months postpartum, sixteen were aged 23–33 years and seven were 34–43 years. Four women had resided in Australia for less than 2 years, 13 for 3–5 years, and six for 6–11 years. Women reported 10 different countries of birth, most commonly Nepal (*n* = 8), Bangladesh (*n* = 3), Indonesia (*n* = 3), Thailand (*n* = 2), and India (*n* = 2). Women identified with 10 different ethnic groups, the main groups were Nepali (*n* = 6), Asian (*n* = 4), and Hindu (*n* = 3). All women spoke a language other than English at home, the main languages spoken were Nepali (*n* = 8), Bengali (*n* = 4), Indonesian Bahasa (*n* = 3). Most women were primiparous (*n* = 21), for the other two women it was their first pregnancy in Australia. The number of interactions with the CCW Service ranged from three to seventeen; two women had five or less, seventeen women had 6–10 and four women had 11–17 interactions.

**Figure 2:**
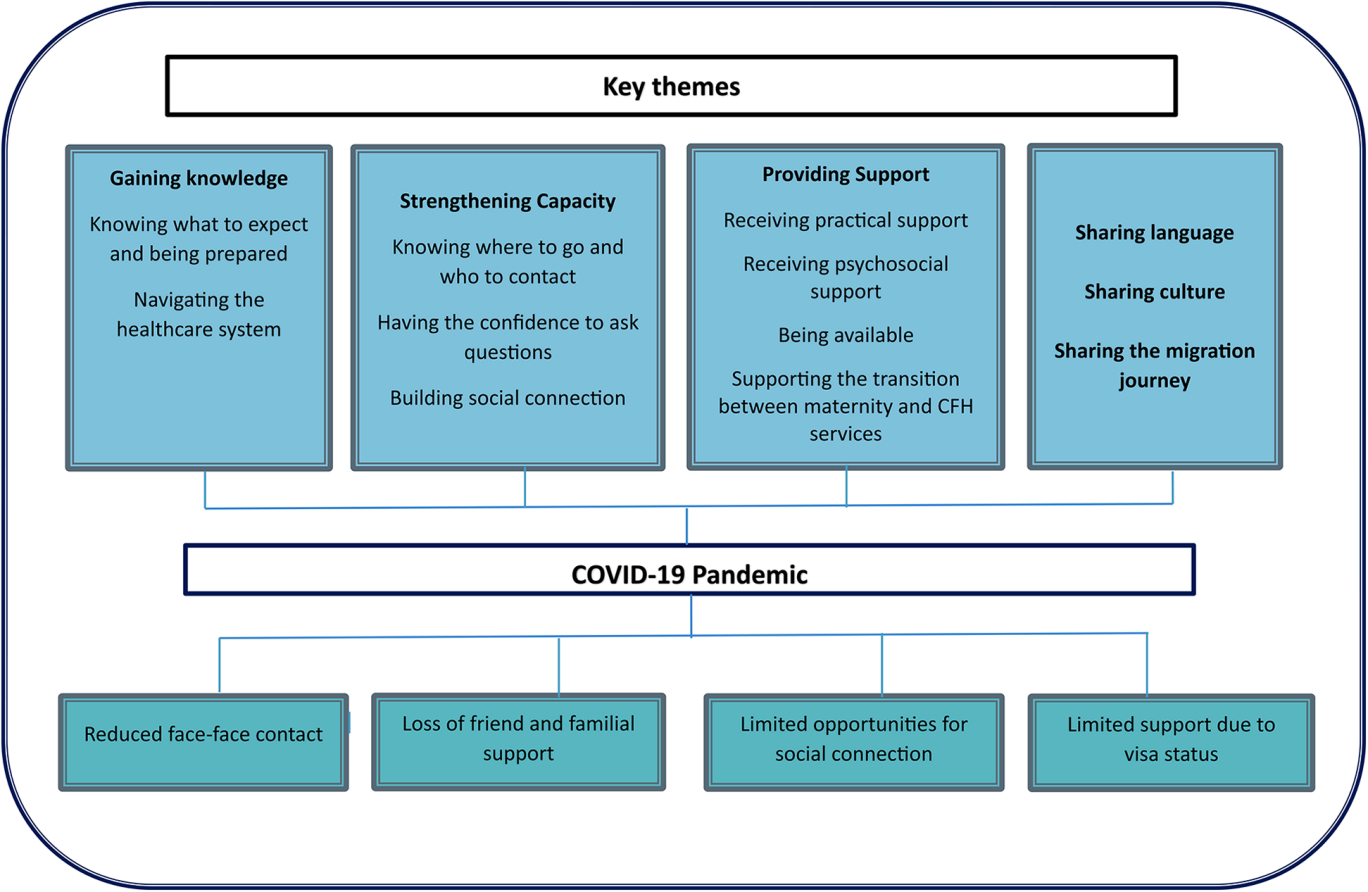
The themes, sub themes from the interviews with women.

Four themes, with associated subthemes were generated: Gaining knowledge, strengthening capacity, providing support sharing language, culture and migration journey. Themes and subthemes are illustrated in Figure 2, including the influence of the COVID-19 pandemic on the themes.

### 3.1 Theme 1: Gaining knowledge

For many women, knowledge was gained through both group and individual education and information sessions during pregnancy and postpartum. In response to the COVID-19 pandemic, most sessions changed from face to face to online. Additionally, COVID-19 restrictions and government-travel restrictions meant many women did not have easy access to information that may have been provided by their family and friends. Subthemes included, knowing what to expect and being prepared, and navigating healthcare systems.

#### 3.1.1 Knowing what to expect and being prepared

Women shared their experience of learning about pregnancy, birth, and parenting, through individual CCW interactions and/or group sessions co-facilitated by the CCW with midwives or CFHN. In particular, information sessions with their partner were valued, for example:

Because it was our first pregnancy, it was very, very different for us and especially from the background that we come,…men from our background, they know nothing about pregnancy at all….so, for me and my husband especially, it was a very, very, very beautiful experience, he appreciated a lot and so did I, that throughout our pregnancy, we got an amazing amount of support, amazing amount of knowledge, information that we never knew, it was amazing (W1, 6 months postpartum).

The education provided by their CCW Service and the midwives meant they knew what to expect as explained here:

I personally feel it would have been bad or worse if I wouldn't come across the CCW…we had no clue of what to do and what not to do… CCW and midwives changed [my] perspective. That was the reason why I worked throughout my pregnancy until 37 weeks and I was perfectly fine…because I came across this cross-cultural thing (W8, 12 months postpartum).

Another woman highlighted the extra information provided by the CCW:

The [CFH] clinic … just mention about [playgroup] but they not really give me a proper address and this kind of thing. They just say, “oh you can find this through the internet” … they promise me that they will put me in the mothers’ group, but no one really do it for me. [CCW] … provides me the address, the telephone, it's clearer. If no CCW, I think that would be challenging because I try to look at the playgroup … from the internet and it's not that clear for me. (W14, 12 months postpartum).

#### 3.1.2 Navigating the healthcare system

The ability to understand and navigate healthcare systems, frequently quite different to their home country, was valued. This included explanation of different clinical roles and responsibilities, scheduling of appointments and interpreters, was expressed by:

I feel like when I met [CCW], I met a sister and good friend…If something not right, I already think about her and she is going to help me… Because when I go to [CCW] and she introduced me to this person and that person…she knows everything in the hospital, like this doctor is for the kids, all the information that I wouldn't get if I had not met her (W6, 12 months postpartum).

A number of women described how the CCW enhanced communication with clinicians which facilitated the navigation of the system, for example:

It would have been so difficult, because even now she helps with my communication and in the hospital with the doctors. [CCW] supported me and I know whatever needs I have for the baby's health; I can always go to her and I know that she would help me or tell me where to go. [CCW] is so great (W12, 6 months postpartum).

### 3.2 Theme 2: Strengthening capacity

The ability of the CCW Service to strengthen women's capacity to support and inform current and future decision-making was valued. Individual and group education sessions with the CCW raised awareness of services to support/enable women's health-seeking. The support of, and trust in the CCW, also increased women's confidence to ask other healthcare/service providers questions and seek information. Most women described the benefits of information sharing within a group setting, enabling them to connect, and develop relationships with other women and families. Components of this theme were: knowing where to go and who to contact, confidence to ask questions, and social connection with other mothers and families.

#### 3.2.1 Knowing where to go and who to contact

The CCW Service supported women's understanding and capacity to access appropriate healthcare and community-based services, including where to go if they were concerned about their own, or their baby's health. In many instances, women had no prior knowledge regarding appropriate services, for example:

[CCW] helped me to make plans for [baby], for routine things, so I would know what to do. In Australia, what type of services here, I did not know this before. I think through [CCW] I know and I got many phone numbers, emergency contact I can call, and I can visit the group and check with the CFHN, I got the information from [CCW] (W5, 12 months postpartum).

#### 3.2.2 Having the confidence to ask questions

Settlement in a new country, limited English proficiency, health literacy, understanding the healthcare system, being informed about perinatal care, and feeling confident to ask questions, enhanced experience and satisfaction with care. Women described feeling safe and confident to ask the CCW questions as CCW interactions were not time-limited and women did not feel rushed. As expressed by:

We feel a bit hesitant to ask some of the questions to GP, but with [CCW] and in the CFH clinics, we can put our questions, so is far better. The main thing is the time. When we visit to GP, sometimes we have a headache with waiting for 3 h with the baby. I feel safe through [CCW] Service. Very thankful for them, rather than visiting GP all the time, more able to ask questions of CCW (W23, 12 months postpartum).

One woman described the CCW as a representative of non-English speaking women and being the appropriate person to advise on available services and supports rather than clinicians:

[CCW] like a frontline representing women who don't speak English as their first language because actually many of those questions, they don't actually need to ask doctor or midwives, but simply…how to access other services like how to book their appointment or something like that, so [CCW] job is very valuable apart from the doctors and nurses’ job. A lot of questions can be answered by [CCW] like they don't need to talk to the doctors for things like finance, or beds or other things like insurance (W18 with interpreter, 6 months postpartum).

The continuity of care meant women trusted the CCW and felt confident and safe to ask questions. This was highlighted by:

[CCW] is kind and very friendly, and helped me… I have things that I am not really clear and then she asked me and provide me information…"I know if something not really clear, I can call her and she can help me to find out. (W17, 6 months postpartum).

#### 3.2.3 Building social connection

The opportunity for social connection with other women and families was appreciated. This was facilitated by the CCW Service through invitations to attend group education sessions, and the CCW sharing information about local community networks, and supported playgroups. One mother described how during a group pregnancy education session the CCW described the importance of social connection with other participants:

You can share everything with a new mum, so it will be good for your baby, they can have friends as well and you can go to the park and walk together, for your mental health as well, [CCW] advised, and after that we became friends (W15, 6 months postpartum).

Many women described the formation of meaningful friendships, support networks, and a community of mothers to share their parenting experience. One mother expressed how the opportunity to connect with others was facilitated by the CCW:

[CCW] organised a video call with the midwife from the hospital with a translator, so all the mums that attended this video call created a [WhatsApp] group, so we could discuss about pregnancy and once we have the baby about the vocational things about babies …we met again during pregnancy and it was really nice. I think it was really helpful, especially to get to know new mums with the same situation (W20, 6 months postpartum).

Another mother expressed how the social connections formed in the face-to face pregnancy group sessions were maintained online during COVID-19 lockdowns:

When I joined the class, [CCW] is very polite and very kind. She was so helpful and she give us the information … and it's really good to meet other friends from the groups. … Around 4 or 5 of us, we are still in touch, we have a group like in Messenger, so we discuss everything on this, so it is very helpful. It's really nice, we had our baby together and now it's lockdown and we can't see each other, but at least we are in touch (W16, 6 months postpartum).

At the time of interviews with women, COVID-19 restrictions further limited social movement and the opportunity for social connection since supported playgroups and community-based groups ceased provision of face-to-face groups, and local Settlement Services or CFHN services had minimal capacity to co-facilitate online groups. This highlighted the importance of social connection, particularly postpartum:

I think motherhood, especially the first few months are very isolating, given the times that we are living maybe even more, so a service that mums from overseas could share experiences. I know it's a bit hard now but maybe do a video call with mums how to deal or cope with motherhood during COVID. … I know it's hard for everyone, … but not having family or friends around, it's even harder sometimes and this is what I missed a little bit. Probably the same way [CCW] did during pregnancy. It could be nice to do follow-up with how everything is going after giving birth (W20, 6 months postpartum).

### 3.3 Theme 3: Providing support

Women valued the CCW Service support, especially as they were managing multiple stressors due to the COVID-19 pandemic (e.g., moving to more affordable housing, loss of their own or their partner’s employment, and inability to access government financial support due to visa status) that impacted on their pregnancy and parenting journey. Women expressed ambivalence about return to their home country, grief with loss of family and friends due to COVID-19, anxiety if friends or family were well, and guilt as to the severity of COVID-19 in their home country compared to Australia. Most women spoke of cancelled family visits due to COVID-19 travel restrictions, and loss of support which might otherwise have been available. Components of this theme were: practical support, psychosocial support, being available, and supporting transition between maternity and CFH services.

#### 3.3.1 Receiving practical support

Practical support provided by the CCW Service had many facets, including knowledge of how to take care of the baby, where to buy affordable baby items, access to free baby and household items from local charities, and agencies providing assistance to families experiencing financial issues. As described:

The [CCW] information was very practical. You can just go to this store and get it, this costs like this much, and you can get that part from the health insurance. It was pretty useful. [The CCW] helped me to find a bit more affordable versions of the services I need (W11, 6 months postpartum).

Another woman explained how the CCW understood the challenge of attending appointments when reliant on public transport and supported her to access a CFHN clinic which was closer to her home:

[CFHN] clinic was recommended by the midwife at the hospital, the midwife arranged the appointment. She only went there twice because it's a bit hard from where she lives and then [CCW] asked her “you want somewhere closer?” and she managed to book the clinic which is much easier and she can take a tram. (W18 with Interpreter, 12 months postpartum).

#### 3.3.2 Receiving psychosocial support

A core component of the relationship between women and the CCW was the provision of psychosocial support; women reported this as the CCWs genuine interest in their wellbeing, that provided a sense of feeling supported, encouraged, and not judged:

The first impression was very good because she is so friendly, she understands me, and I can talk about my problems, or my curiosity, or my anxiety to her and no judgement. She helps me and gives me advice and I can call her any time. …every time she will ask about my situation or how I am feeling and I mean she always follow-up about my feeling, or my situation, or my problems, that's really helpful (W19, 6 months postpartum).

Many women spoke of COVID-19 pandemic restrictions reducing face-to-face accessibility to practical and emotional support from family and friends internationally, nationally, and locally. In the absence of familial support, the CCW was a highly valued source of psychological support and advice. Women described the CCW as a trusted nonjudgmental family member, who enabled them to feel heard and supported:

When you have no one, your close friends, family, parents, sister, brother, you can't call every day and for every single thing and tell them, so here I think for us, that was our family, so we can share all the things that I feel that way (W21, 6 months postpartum).

#### 3.3.3 Being available

An important aspect of the CCW Service highly valued by women was the availability, accessibility and timely response to questions and issues as they arose. This aspect of being available was expressed by:

Best part was I can call anytime for any help, she always answers her phone, and she always listens carefully what I am saying, and she always asks questions … she wants to understand what I am feeling or what I really want to know … she's there for you, but maybe it's not about the baby and all, but just gives me time (W3, 6 months postpartum).

However, some women expressed challenges contacting the CCW due to the part-time nature of the role and no backfill during CCW leave, for example:

[CCW] used to work two or three days or something like that in the hospital, if she is not working, have another person that replace her or maybe when she goes on holiday, … because if not, the women probably need her for some information and she wasn't there. …maybe not [CCW] working full time, but at least have someone to stand by (W14, 12 months postpartum).

#### 3.3.4 Supporting the transition between maternity and child and family health services

Several women spoke of the CCWs role in supporting the transition from maternity to community-based services, including breastfeeding support, CFHN, Women's Health nurses, General Practitioners, postpartum groups, and supported playgroups. In particular, women appreciated reminders to attend infant health and development checks with CFHN, and infant immunisations with General Practitioners:

[CCW] keeps reminding about the vaccinations and the last time she rang me for the COVID vaccination booking as well. Once I forgot to book an appointment for 6-month check-up and she reminded me…And the playgroup which is run by the councils and the libraries, she suggests me to take my baby there for the playgroup so that she can observe and she could socialise (W13, with Interpreter, 12 months postpartum).

### 3.4 Theme 4: Sharing language, culture and migration journey

Women appreciated being able to speak to the CCW in their first language, and the availability of in-language information. Additionally, women described when there was no shared language, the CCW recognised the need to speak slowly using plain English, and allow time for women to ask questions to ensure clear understanding.

Women acknowledged the benefits of shared culture as the CCW enquired about preferred cultural practices and shared these with clinicians. Shared experience of the migration and settlement journey were also highly valued aspects of CCW role as they provided advocacy in regard to cost of maternity care, and recognised the financial impact of the COVID-19 pandemic on their communities.

#### 3.4.1 Sharing language

The CCW Service was able to address language issues for some women, for example:

They [CCW] are really helpful, because in our community, girls have so many problems with the language and their communication skills, so it's really helpful for them, because they can talk in their language. When your language is not good, with the doctor as well, you want to say and ask so many things, but for the language, I think it is a barrier to you, but in that kind of a service you have, what you can't say to your doctor, you can say to [CCW] because of your language, then she can really help you in many ways (W22, 6 months postpartum).

One woman described that, although the CCW did not share the same language, her accent enhanced clarity and understanding of the information provided, and encouraged women to share experiences:

She is doing very good job. … For Asians the accent of the Americans and Australians is a bit hard to understand sometimes, some of the words, but the way [CCW] is explaining I mean her accent, the way she talks is so clear, it makes it so easy to understand and I think for everyone … she is very easy going, so it's easy to open up the problems with her (W23, 12 months postpartum).

The absence of shared language was a barrier to accessing the CCW Service, particularly for women who may be nervous to converse in English:

Maybe they feel that [CCW] doesn’t speak Thai, so anything has to be done through English, …also they do not want to share, so they wait and wait to read the Thai forum on Facebook instead of talking directly to [CCW] or to a doctor…. They fear that they may not understand [CCW] or doctor when they are speaking. If there is a multicultural lady like [CCW] but speaks Thai,….the Thai ladies would be more confident in accessing the service (W18 with Interpreter, 6 months postpartum).

#### 3.4.2 Sharing culture

Shared culture was acknowledged in terms of role expectations of partners, supporting cultural practices and preferences. One woman described how the CCW had visited her home country and had awareness of some of the cultural customs and asked if she wanted to practice these preferences during her pregnancy:

I think [CCW] told me she's been to Mongolia…that was nice for me to hear. That someone else has been to my place, knew some customs and enjoyed it there (W21, 6 months postpartum).

#### 3.4.3 Sharing the migration journey

The CCWs lived experience of migration and the settlement journey enabled recognition of the barriers women and families may encounter when accessing services. One woman described how her partner felt that the purpose of a CCW visit on the postnatal ward was to ensure they received satisfactory care:

[Partner] said the [CCW] came to visit and asking if any concern, how we are going, and making sure we are okay. We get all the facilities and check if midwife is helpful and things like that, because sometimes we don't speak…the [CCW] is there to make sure that we get the same treatment (W15, 12 months postpartum).

Several women described the challenges of pregnancy and parenting in a new country alongside the impact of the COVID-19 pandemic, and expressed gratitude for the continuity of care and support provided by the CCW Service, for example:

Sometimes the midwives or nurses don’t know what you are going through, because you are from another country. I don't know living your pregnancy without your family and not speaking English very well, the fact that you know someone who gives you information and is aware of your situation, it really helps you, especially if it's your first pregnancy. … having this service where you could talk with someone and feel a bit relaxed, it was really helpful. (W20, 6 months postpartum).

## 4 Discussion

This study found that the CCW Service was highly valued by women and seen as helpful, informative and supportive. Integral components reflected in the themes were provision of support and gaining knowledge, strengthening capacity, and sharing language, culture and lived experience of the migration journey. Interacting and impacting all these areas was the COVID-19 pandemic. The CCW Service was perceived to be effective in provision of practical and psychosocial support and facilitating the transition from maternity to CFH services. This ensures ongoing service engagement to support child health and wellbeing, which is known to decline in families from migrant and refugee backgrounds (18, 26). Suggestions for CCW Service improvement from women, included increased CCW workforce to enable timely response to questions, and increased provision of group education to support information sharing and opportunities for social connection in the postpartum period.

Women in our study reported high levels of satisfaction with the CCW model, aligning with our prior women and partner surveys and service provider surveys and interviews (23, 27). While earlier work established that CCWs effectively support access to information and education (23, 27), this study deepens our understanding by identifying specific mechanisms through which CCWs achieve this—particularly through their lived experience of migration, shared language, and cultural understanding. While earlier research identified CCWs' cultural understanding as important (23, 27), our current study demonstrates how this cultural responsiveness specifically enables continuity of care and supports women's confident, independent service engagement. Our study provides new insights into women's perinatal needs, particularly highlighting the value of group education for both information sharing and social connection—an aspect not fully explored in previous work. The workforce challenges identified in our study echo and strengthen previous findings (23, 27). However, our current study adds nuance by linking workforce capacity directly to timely response capabilities and group education—two specific areas that women identified as crucial for service improvement. This alignment across multiple studies, while identifying new dimensions, underscores the need for workforce expansion to meet growing service demands and maintain quality across both maternity and CFH services.

These findings also align with a recent systematic review of models of care (19), which identified nine studies that evaluated the impact of bicultural/bilingual/multicultural/peer workers in perinatal service provision (28–36). The CCW Service engaged similar approaches reported in the literature, including continuity of care (29, 31, 33), information sharing (28, 31, 33–36), provision of social support (28, 31, 33, 36), enhanced communication between women and service providers (28, 31, 33), and cultural sensitivity of service providers (28–36). Recent studies further highlight the benefits of bicultural/bilingual/multicultural/peer workers, including community-based group pregnancy care for women from refugee backgrounds (37, 38).

The impact of the COVID-19 pandemic was significant in this study, including the loss of friends and familial supports that would otherwise have been available except for imposed travel restrictions (7, 39–42). There were also changes in service provision, including limited face-to-face interactions with clinicians and the CCW Service, and disrupted face-to-face education, community support groups and networks, and supported playgroups, which would otherwise have supported social connection between mothers and families. Although women were positive about their interactions with health services, it was evident services were not always responsive to issues related to settlement in a new country, the stressors associated with COVID-19 particularly for families from migrant and refugee backgrounds, alongside the transition to parenting.

Loneliness in pregnancy and the postpartum period has been reported particularly for parents isolated from familial and cultural connections with others who shared their values and practices (43) especially during the COVID-19 pandemic (7, 39–41). Protective of loneliness in families from migrant and refugee backgrounds was the presence of family and friends, especially female family members for mothers (44). Some women in the study expressed gratitude for the CCW Service being available to substitute the practical and psychosocial support otherwise obtained from familial supports especially from those who have experienced similar situations.

The complexity of navigating health services, particularly the transition between maternity and CFH services, are key barriers to access for women and families from migrant and refugee backgrounds (16). Bicultural/bilingual workers have demonstrated their positive contribution to improving access to maternity and CFH services through navigation support (23, 27, 28, 32, 33, 37, 45, 46), fostering relationships between service providers and service users to improve the cultural responsiveness of services (23, 27, 32, 37, 47). Their support and education can increase health literacy, reinforce the importance of maternal and CFH health, and child developmental assessments, in order to optimise health outcomes (16, 48).

The CCW Service was highly valued regarding knowledge acquisition including discussing the transition to parenting and mental health issues, challenges of breastfeeding and its importance, and enhanced by groups being face-to-face vs. online. However, some women felt the CCW Service could be improved through the provision of more education groups, particularly with a focus on mental health and financial issues. This may align with the COVID-19 pandemic whereby migrant and refugee communities were significantly impacted by loss of employment, separation from and loss of family supports during an already significant time as transitioning to parenthood.

One strength of this study is the deeper understanding of women's perspectives of the CCW Service at 6 and 12 months postpartum using semi-structured interviews. A further strength of the study was that the interviews were undertaken with an interpreter when required. Limitations include that due to the interview period coinciding with COVID-19 pandemic lockdowns, many women were over-burdened, and consequently less likely to participate in interviews, with approximately one-third of those selected not participating. Women who consented to participate may have had been more likely to have a positive experience with the CCW Service. Participants may also have been hesitant to voice dissatisfaction or concerns about their care and the CCW service, even though they were assured prior to interview that their perspective would have no impact on the CCW's employment or on their own current or future care. Cultural factors and power dynamics may have influenced participants' willingness to express criticism. This could include cultural values emphasizing respect for healthcare providers, feelings of gratitude for receiving support, or concerns about maintaining harmonious relationships within their communities. Additionally, women who discontinued engagement with the CCW Service or had negative experiences may have been less likely to participate, limiting our understanding of service barriers or challenges. While our methodology included specific measures to encourage open dialogue and critical feedback, these inherent limitations should be considered when interpreting the findings. However, we are cognizant this approach has the potential for respondent bias and favorable feedback. Future research would benefit from targeted strategies to engage with women who declined or discontinued CCW support to capture a broader range of experiences. Studies have reported that participation may be enhanced through involvement of trusted workers (28, 49).

Another possible limitation that may influence findings, is the lead researcher (HJR), who implemented and is also the manager of the CCW Service. As detailed in Materials and Methods, the lead researcher undertook multiple reflexive and self-inquiring practices including journalling and regular discussion with co-authors to minimize potential bias.

This study highlights how health services can provide an individualised approach that responds to the complex perinatal needs of women and families from migrant and refugee backgrounds. The presence and support of bicultural/bilingual workers helped establish cultural and emotional safety for women, improved communication between women, their families, and health professionals, supported women's antenatal and postpartum needs, and enhanced women's ability to make health decisions for themselves and their children to improve health outcomes *(*47). Our findings support the Australian Practice Guidelines for Pregnancy and Postnatal Care (49) and World Health Organization guidelines (5) that recommend models of care that involve bicultural/bilingual workers to ensure culturally responsive service provision.

The findings have the potential to support scalability and sustainability in HIC with adaptations based on local settings and communities. Migrant women and families are not a homogenous group, so workers with shared language and culture may be required to support local priority populations, and needs must be carefully considered to avoid “othering” of women into risk groups (50). Adaptations also require conversations and co-design with local migrant and refugee communities to understand socioeconomic and cultural factors, health beliefs and practices that impact on service access (19).

## 5 Conclusion

The CCW Service was highly regarded by women who perceived it to be supportive, helpful and informative, which enhanced their perinatal experience and perception of care. Women appreciated the CCW Service supporting them to gain knowledge to prepare for pregnancy, birth and parenting, inform current and future decision-making, navigate perinatal health services, information sharing within a group setting enabled social connection with other women and families, reminders to attend CFH services. We recommend further research to strengthen understanding and implementation of targeted models to assist healthcare providers and policymakers in resource allocation decisions.

## Data Availability

Datasets are available from the corresponding author on reasonable request. Requests to access the datasets should be directed to h.rogers@student.unsw.edu.au.
